# Impacts of *ADH1B* rs1229984 and *ALDH2* rs671 polymorphisms on risks of alcohol‐related disorder and cancer

**DOI:** 10.1002/cam4.4920

**Published:** 2022-06-06

**Authors:** Ting‐Gang Chang, Ting‐Ting Yen, Chia‐Yi Wei, Tzu‐Hung Hsiao, I‐Chieh Chen

**Affiliations:** ^1^ Department of Psychiatry Taichung Veterans General Hospital Taichung Taiwan; ^2^ School of Psychology Chung Shan Medical University Taichung Taiwan; ^3^ Department of Otorhinolaryngology Taichung Veterans General Hospital Taichung Taiwan; ^4^ School of Medicine National Yang Ming Chiao Tung University Taipei Taiwan; ^5^ Department of Medical Research Taichung Veterans General Hospital Taichung Taiwan; ^6^ Department of Public Health, College of Medicine Fu Jen Catholic University New Taipei City Taiwan; ^7^ Institute of Genomics and Bioinformatics National Chung Hsing University Taichung Taiwan

**Keywords:** *ADH1B* rs1229984, alcohol‐related cancer, alcohol‐related disorder, *ALDH2* rs671

## Abstract

**Background:**

*ADH1B* rs1229984 and *ALDH2* rs671 are the specifically prevalent functional variants in the East Asians. These variants, which result in a dramatic change in enzyme activity, are highly associated with alcohol‐related disorders and cancer. Previous studies focusing on the additive and synergic effects of the variants are few and inconsistent. The aim of the research was to evaluate the associations of *ADH1B* rs1229984 and *ALDH2* rs671 with the risks of alcohol‐related disorder and cancer.

**Methods:**

This cohort study enrolled 42,665 participants from the Taiwan Precision Medicine Initiative database, including 19,522 and 20,534, *ADH1B* and *ALDH2* carriers, respectively. The associations between the two variants and cancer risk were analyzed by univariable and multivariable logistic regression.

**Results:**

Compared with the noncarriers, the *ADH1B* rs1229984 variant had a stronger effect on alcohol‐related disorders and was related to an increased risk of alcohol‐related cancers. The CC genotype of *ADH1B* rs1229984 was significantly associated with cancer of the larynx, pharynx, and nasal cavities [odds ratio (OR) = 1.56, *p* = 0.0009], cancer of the pancreas (OR = 1.66, *p* = 0.018), and cancer of the esophagus (OR = 4.10, *p* < 0.001). Participants who carried the rs1229984 TC/CC and rs671 GG genotypes were at higher risk of esophageal cancer (OR = 3.02, *p* < 0.001). The risk of esophageal cancer was increased by 381% (OR = 4.81, *p* < 0.001) in those carrying the rs1229984 TC/CC and rs671 GA/AA genotypes.

**Conclusion:**

rs1229984 and rs671 are common and functionally important genetic variants in the Taiwanese population. Our findings provide strong evidence of additive and synergic risks of *ADH1B* and *ALDH2* variants for alcohol‐related disorders and cancer. The results suggested that are reduction in alcohol consumption should be advised as a preventive measure for high‐risk patients carrying *ADH1B* rs1229984 C or the *ALDH2* rs671 A allele.

## Graphical abstract

Figure. The Flow char for selection of the study population (A), and the PheWAS Manhattan and QQ plots for ADH1B rs1229984 (B) and ALDH2 rs671 (C), respectively Brief Summary Our findings provide evidence of additive and synergic risks of the ADH1B and ALDH2 variants for alcohol‐related disorder and cancer. These results suggested that reduction of alcohol consumption should be advised as a preventive measure for high‐risk patients carrying the ADH1B rs1229984 C or the ALDH2 rs671 A allele.

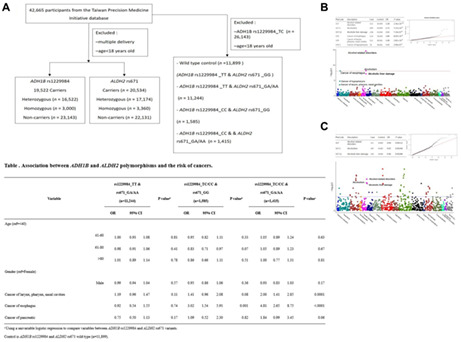

## 1 INTRODUCTION

Alcohol consumption is a crucial factor in excess of 200 diseases and injuries. It is related to a risk of developing health problems such as alcohol dependence, cancers, cardiovascular diseases, and violence.^1^, ^2^ Before the publication of the Diagnostic Statistical Manual of Mental Disorders, 5th Edition (DSM‐5), alcohol issues were generally divided into abuse and dependence. Alcohol abuse can be defined as recurrent social, interpersonal, health, and legal problems as a result of alcohol use. Alcohol dependent referred to alcohol tolerance, withdrawal symptoms, drinking to relieve or avoid withdrawal symptoms, and awareness of the compulsion to drink or crave alcohol. The DSM‐5 combines these categories into a single substance use disorder, measured on a continuum from mild to severe.

Multifactorial inheritance is when more than one factor causes a trait or health problem, such as alcohol use disorder and alcohol‐related cancers. The main factor is genes. However, the cause includes other factors that are not genes, such as alcohol. Past literature has investigated the gene–environment interaction between alcohol consumption and the aldehyde dehydrogenase 2 (ALDH2) genotype in upper aerodigestive tract cancer in Japan and found a statistically significant gene‐environment interaction for alcohol consumption and the *ALDH2* genotype.^3^ Therefore, polymorphisms are strongly influenced by environmental conditions, to reach a final phenotype related to the presence of disease.

Since 1988, alcoholic beverages have been classified as group 1 carcinogens to humans by the International Agency for Research on Cancer (IARC)^4^ based on evidence of the toxicity of ethanol from epidemiological and animal studies.^5^ In 2010, IARC classified ethanol, the major constituent of alcoholic beverages, and its metabolite acetaldehyde as carcinogenic to humans.^6^ According to a recent study, an estimated 3.2% of cancer deaths in the United States (approximately 18,947 deaths) are alcohol‐related.^7^ Globally, an estimated 741,300 (95% uncertainty intervals 558,500–951,200) or 4.1% (3.1–5.3) of all new cases of cancer in 2020 were attributable to alcohol consumption.^8^ Researchers have proposed several ways that alcohol may increase the risk of cancer, such as metabolizing alcohol to acetaldehyde, acetaldehyde can damage both DNA and proteins^9^; generating reactive oxygen species, which can damage DNA, proteins, and lipids in the body through oxidation^10^; impairing the body's ability to breakdown and absorb a variety of nutrients that may be associated with cancer risk; and increasing blood levels of estrogen.^11^ Alcohol may also indirectly contribute to cancer development by acting as a solvent for other carcinogens, such as chemicals in tobacco.^12^

Alcohol metabolism involves the functional variants alcohol dehydrogenase 1B (*ADH1B*) rs1229984 and aldehyde dehydrogenase 2 (*ALDH2*) rs671, which are specifically found in East Asian populations.^13^, ^14^, ^15^ The frequencies of the rs1229984 C allele and rs671 A allele are 0.262 and 0.255 among East Asians, respectively, but these are rare or occur at low frequencies in other populations (frequency = 0.047 and 0.0003, respectively).^16^ In the Taiwanese population, approximately 47% of people carry the A allele of the *ALDH2* rs671, with decreased enzyme activity.^13^ Similarly, approximately 50.5% of the people carry the C allele of *ADH1B* rs1229984.^13^ *ALDH1B1* shares a 75% peptide sequence homology with *ALDH2*.^17^

Genetic polymorphisms of *ADH1B* and *ALDH2* lead to changes the enzyme activation in alcohol metabolism, affecting the accumulated level of acetaldehyde after alcohol intake. The *ADH1B* rs1229984 T allele and *ALDH2* rs671 A allele have been found to be associated with acetaldehyde accumulation after drinking alcohol. ADH1B is a member of a family of alcohol dehydrogenases that facilitates the interconversion between alcohols and aldehydes or ketones with the reduction of NAD^+^ to NADH. ALDH2, encoded by the *ALDH2* gene on chromosome 12, belongs to the aldehyde dehydrogenase gene family and catalyzes the oxidation of aldehydes to their corresponding acids.^18^ Acetaldehyde is related to the unfavorable symptoms associated with alcohol consumption, such as palpitations, nausea, and headache. A previous study that investigated alcohol ingestion in a Chinese population according to the *ALDH2* rs671 polymorphism revealed that individuals carrying the GA genotype consumed less than 17 g of alcohol per day.^19^ The inactive ALDH2*2 (+) genotype (*1/*2 or *2/*2), resulting in acetaldehyde accumulation, provides a protective effect against alcohol use disorder.^20^ The *ADH1B* rs1229984 T allele causes a rapid conversion of ethanol to acetaldehyde and leads to an aversive reaction to alcohol and a protective effect against alcohol use disorder.^20^, ^21^, ^22^ Individuals with the *ADH1B* rs1229984 (TC) or rs1229984 (TT) genotypes are more likely to experience drinking discomfort, reducing the risk of alcohol use disorder.^14^, ^23^ Previous studies have revealed protective roles of the ADH1B*2(+) and ALDH2*2(+) genotypes against the development of alcohol dependence.^24^ However, the additive and synergic effects among*ADH1B* rs1229984 and *ALDH2*rs671 on alcohol addiction have been unclear until now.

Alcohol is considered a carcinogen with a clear dose–risk relationship.^25^ According to an association study of *ADH1B* and *ALDH2* polymorphisms,^26^ East Asian populations may be more susceptible to the carcinogenic effect of alcohol, including esophageal cancer and head and neck cancer.^26^ Genetic variants involved in alcohol metabolism and detoxification are also thought to affect susceptibility to alcohol‐associated cancers. However, the reported risks of alcohol‐associated cancers related to *ADH1B* rs1229984 and *ALDH2* rs671 in East Asians are inconsistent, and heterogeneity in distinct studies has been substantial.^27^Here, we performed a large‐scale hospital‐based case–control study to explore the associations of *ADH1B* and *ALDH2* variants with the risks of alcohol‐related disorder and cancer.

## 2 MATERIALS AND METHODS

### 2.1 Data sources

This retrospective hospital‐based case–control study included 42,665 Taiwanese from the Taiwan Precision Medicine Initiative (TPMI), which gathered electric health record information and specimens from participants in Taichung Veterans General Hospital (TCVGH, Taichung City, Taiwan) from June 2019 to June 2020. Participants aged >18 years who visited 28 medical and surgical outpatient clinics in TCVGH were invited to participate in the TPMI study. Data with demographics, medical history, genotyping information, and biochemical reports were obtained from the TPMI. The research protocol was conducted in accordance with the Declaration of Helsinki.

### 2.2 Participants

This study involving human participants were approved by the ethics committee of Taichung Veterans General Hospital Institutional Review Board (IRB no. SF19153A), and all of the participants provided written informed consent. We selected participants for whom genotyping information for *ADH1B* rs1229984 and *ALDH2* rs671 was available; 23,143 participants with the TT genotype of *ADH1B* rs1229984 were defined as *ADH1B* rs1229984 variant noncarriers, and 22,131 participants with the GG genotype of*ALDH2* rs671 were defined as *ALDH2* rs671 variant noncarriers. Finally, 16,522 participants with the *ADH1B* rs1229984 variant (genotype TC) were defined as *ADH1B* rs1229984 heterozygous variant carriers, and 3000 participants (genotype CC) were defined as *ADH1B* rs1229984 homozygous variant carriers; regarding *ALDH2* rs671 carriers, 17,174 (GA genotype) and 3360 (AA genotype) participants were defined as *ALDH2* rs671 hetero and homozygous variant carriers, respectively. We then compared the association between *ADH1B* and *ALDH2* polymorphisms and the risk of cancers. There were 11,899 participants with the *ADH1B* rs1229984 TT genotype and *ALDH2* rs671 GG genotype who were defined as the control group, they only had *ADH1B* and *ALDH2* wild‐type genes. Participants carrying the *ADH1B* rs1229984 TT and *ALDH2* rs671 GA/AA genotypes (*n* = 11,244), *ADH1B* rs1229984 TC/CC and *ALDH2* rs671 GG genotypes (*n* = 1585), and *ADH1B* rs1229984 TC/CC and *ALDH2* rs671 GG/AA genotypes (*n* = 1415), defining as case group, respectively (Table 6).

### 2.3 Genotype generation

The customized array‐TWBv2 was designed based on the GRCh38 coordinates and employed whole‐genome sequence (WGS) data from the Taiwan Biobank (TWB). These data for the researchers to select single nucleotide polymorphisms (SNPs) for the best imputation for the Han Chinese samples that were collected in Taiwan. This array includes 114,000 risk variants in 2831 unusual disease genes chosen from the previously published literature.^28^ In this study, we genotyped 42,665 participants by using the TWBv2 array and calculated the minor allele frequencies of *ADH1B* rs1229984 and *ALDH2* participants without rs1229984rs671 with normalization of the TWB reference panel.

### 2.4 Covariates

The clinical diagnoses in our study were according to the International Classification of Diseases, Ninth Revision, Clinical Modification (ICD‐9‐CM) diagnosis codes, with at least two records of an outpatient diagnosis or one record of an inpatient diagnosis during the period from January 2009 to June 2020. According to the severity, alcohol‐related disorders can be divided into alcohol abuse (ICD‐9‐CM 305), alcohol dependence syndrome (ICD‐9‐CM 303), and alcohol‐induced mental disorders (ICD‐9‐CM 291). The cancer types included are cancer of the larynx, pharynx, and nasal cavities (ICD‐9‐CM 146–149, 160–161), cancer of the esophagus (ICD‐9‐CM 150), and cancer of the pancreas (ICD‐9‐CM 157).

### 2.5 Phenome‐wide association study (PheWAS)

PheWAS was used to duplicate the genetic associations and identify new phenotype associations for genetic variants. In this study, the genetic data analysis and plotting tools for PheWAS was performed using R4.1.1.^29^ Manhattan plots of PheWAS data were utilized to relate phenotype with genotype. The *x*‐axis was displayed various disease groups in distinct colors, while the *y*‐axis showed p values of individual phenotypes. Red and blue horizontal line is represent as the Bonferroni corrected *p* < 5 × 10^−8^ and the significance level of *p* = 0.05, respectively. The quantile‐quantile (QQ) plot was used to determine statistics under the null hypothesis.

### 2.6 Statistical analysis

Clinical data were analyzed using the SAS version 9.3 software (SAS Institute Inc.). Chi‐square test was utilized to calculate the statistical significance between categorical variables. Associations of genotypes with the risks of alcohol‐related disorders and cancers were calculated by logistic regression. Significant covariates were included in the final model; a two‐tailed test *p* value of <0.05 was considered statistically significant in this study.

## 3 RESULTS

Between June 2019 and June 2020, 42,665 patients (men and women) were identified from the TPMI, with a mean age of 55.67 ± 15.249 for the male participants and 57.66 ± 15.322 for the female participants. The basic characteristics of the participants recruited from TCVGH and analyzed by genotyping microarray are shown in Table 1. All participants were identified as carrying*ADH1B* rs1229984 or *ALDH2* rs671 variants related to alcohol metabolism based on the TWBv2 array. Overall, 42,665 patients, 19,522 participants were *ADH1B* rs1229984 C allele carriers (TC/CC genotype) and 20,534 were *ALDH2* rs671 A allele carriers (GA/AA genotype). The ratios of esophageal cancer and alcohol dependence syndrome were 0.97% (*p* < 0.001) and 1.10% (*p* < 0.001), respectively, in the *ADH1B* rs1229984 CC genotype. The ratio of cancer in larynx, pharynx, and nasal cavities was also significantly higher in *ADH1B* rs1229984 CC genotype (2.33%, *p* = 0.004). Ratio of esophageal cancer was higher in *ALDH2* rs671 GA genotype (0.45%, *p* = 0.005) than in the *ALDH2* rs671 noncarriers.

**TABLE 1:** Basic characteristics of the study subjects

Variable	rs1229984 noncarriers TT genotype (*n*=23,143)		rs1229984 carriers TC genotype (*n*=16,522)		rs1229984 carriers CC genotype (*n*=3000)		*p* value^a^	rs671 noncarriers GG genotype (*n*=22,131)		rs671 carriers GA genotype (*n*=17,174)		rs671 carriers AA genotype (*n*=3,360)		*p* value^a^
	*n*	%	*n*	%	*n*	%		*n*	%	*n*	%	*n*	%	
Age							0.78							0.46
<40	3641	15.73	2591	15.68	489	16.30		3424	15.47	2738	15.94	559	16.64	
41–60	8760	37.85	6325	38.28	1166	38.87		8421	38.05	6532	38.03	1298	38.63	
61–80	9360	40.44	6621	40.07	1175	39.17		8959	40.48	6893	40.14	1304	38.81	
>80	1382	5.97	985	5.96	170	5.67		1327	6.00	1011	5.89	199	5.92	
Gender							0.3							0.04
Female	12518	54.09	9013	54.55	1664	55.47		11932	53.92	9378	54.61	1885	56.1	
Male	10625	45.91	7509	45.45	1336	44.53		10199	46.08	7796	45.39	1475	43.9	
Cancer of larynx, pharynx, nasal cavities	352	1.52	260	1.57	70	2.33	0.004	324	1.46	301	1.75	57	1.7	0.07
Cancer of esophagus	56	0.24	63	0.38	29	0.97	<0.0001	66	0.30	77	0.45	5	0.15	0.005
Cancer of pancreatic	94	0.41	60	0.36	20	0.67	0.06	98	0.44	65	0.38	11	0.33	0.46
Alcohol‐induced mental disorders	29	0.13	25	0.15	11	0.37	0.006	58	0.26	7	0.38	0	0	<0.0001
Alcohol dependence syndrome	65	0.28	46	0.28	33	1.10	<0.0001	124	0.56	20	0.12	0	0	<0.0001
Alcohol abuse	42	0.18	32	0.19	9	0.30	0.38	63	0.28	19	0.11	1	0.03	<0.0001

^a^ The statistical significance between categorical variables were analyzed by the Chi‐square test.

**FIGURE 1:**
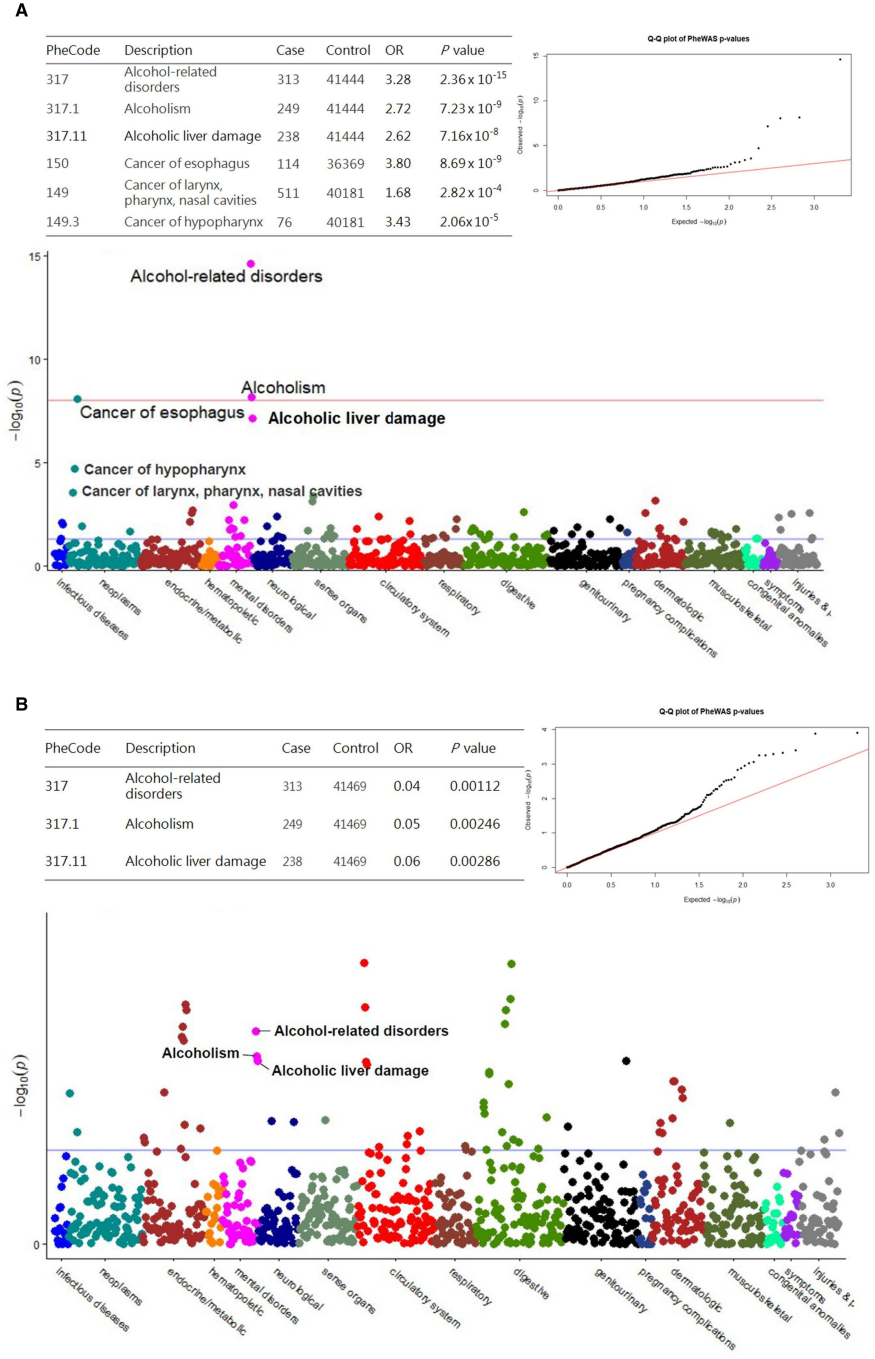
The PheWAS Manhattan and QQ plots for ADH1B rs1229984 (A) and ALDH2 rs671 (B), respectively, among 42,665 participants from the study cohort. The table for the number of case and control per genotype. Along the *x*‐axis different disease groups are shown in different colors and the *y*‐axis reflects the *p* value for each phenotype. Blue and red horizontal line is represented as the significance level of *p* = 0.05 and Bonferroni corrected significant threshold (*p* < 5 × 10^−8^).

Then, we tested the association between SNPs and clinical phenotype with PheWAS. As shown in Figure 1, the Manhattan plots and Q‐Q plots for *ADH1B* rs1229984 and *ALDH2* rs671, respectively. We found that *ADH1B* rs1229984 was significantly associated with a variety of alcohol‐related phenotypes and cancers. The cancers associated with *ADH1B* rs1229984 were in the head and neck region (Figure 1A), including cancer of the larynx, pharynx, and nasal cavities (OR = 1.68, *p* = 2.82 × 10^−4^), cancer of the hypopharynx, (OR = 3.43, *p* = 2.06 × 10^−5^) and cancer of the esophagus (OR = 3.80, *p* = 8.69 × 10^−9^). The association with alcohol‐related phenotypes included alcohol‐related disorder (OR = 3.28, *p* = 2.36 × 10^−15^), alcoholism (OR = 2.72, *p* = 7.23 × 10^−9^), and alcoholic liver damage (OR = 2.62, *p* = 7.16 × 10^−8^). On the other hand, *ALDH2* rs671 was found to be associated with alcohol‐related phenotypes, but not with cancers in the head and neck region (Figure 1B) in this study. *ALDH2* rs671 exhibited a protective role in terms of alcohol‐related disorder (OR = 0.04, *p* = 1.12 × 10^−3^), alcoholism (OR = 0.05, *p* = 2.46 × 10^−3^), and alcoholic liver damage (OR = 0.06, *p* = 2.86 × 10^−3^) (Figure 1B).

Next, we investigated the association between *ADH1B*/*ALDH2* polymorphisms and diseases. As shown in Table 2, univariable logistic regression analysis revealed that the risk of esophageal cancer was significantly associated with the C allele of *ADH1B* rs1229984, and both the TC and CC genotypes contributed to raise risk of esophageal cancer in *ADH1B* rs1229984 carriers (TC: OR = 1.58, 95% CI: 1.10–2.26, *p* = 0.013; CC: OR = 4.02, 95% CI: 2.57–6.31, *p* < 0.001) compared with their counterparts. Additionally, the CC genotype of *ADH1B* rs1229984 is significantly associated with cancer of the larynx, pharynx, and nasal cavities (OR = 1.55, *p* = 0.001), alcohol‐induced mental disorders (OR = 2.93, *p* = 0.002), and alcohol dependence syndrome (OR = 3.95, *p* < 0.001). In contrast, the markedly decreased risk of alcohol‐induced mental disorders (OR = 0.13), alcohol dependence syndrome (OR = 0.17), and alcohol abuse (OR = 0.34) was statistically significant for the *ALDH2* rs671 A allele carriers (*p* < 0.001).

**TABLE 2:** Association between *ADH1B/ALDH2* genotypes and alcohol‐related disorder/cancer

Variable	rs1229984_TC genotype(*n* = 16,522)			*p* value^a^	rs1229984_CC genotype(*n* = 3000)			*p* value^a^	rs671_GA/AA genotype(*n* = 20,534)			*p* value^a^
	OR	95% CI			OR	95% CI			OR	95% CI		
Age (ref = <40)												
41–60	1.02	0.96	1.08	0.50	0.99	0.89	1.11	0.35	0.97	0.91	1.02	0.99
61–80	0.99	0.94	1.06	0.63	0.94	0.84	1.05	0.44	0.95	0.90	1.01	0.34
>80	1.00	0.91	1.10	0.98	0.92	0.76	1.10	0.46	0.95	0.86	1.04	0.53
Gender (ref = Female)												
Male	0.98	0.94	1.02	0.36	0.95	0.88	1.02	0.15	0.96	0.93	1.00	0.05
Cancer of larynx, pharynx, and nasal cavities	1.04	0.88	1.22	0.67	1.55	1.19	2.01	0.001	1.19	1.03	1.39	0.02
Cancer of esophagus	1.58	1.10	2.26	0.013	4.02	2.57	6.31	<0.0001	1.34	0.97	1.85	0.08
Cancer of pancreatic	0.90	0.65	1.24	0.50	1.65	1.01	2.67	0.04	0.84	0.62	1.13	0.24
Alcohol‐induced mental disorders	1.21	0.71	2.07	0.48	2.93	1.46	5.88	0.002	0.13	0.06	0.29	<0.0001
Alcohol dependence syndrome	0.99	0.68	1.45	0.96	3.95	2.59	6.02	<0.0001	0.17	0.11	0.28	<0.0001
Alcohol abuse	1.07	0.67	1.69	0.78	1.66	0.81	3.41	0.17	0.34	0.21	0.57	<0.0001

^a^ Compare variables between the carriers and noncarriers by the univariable logistic regression model.

The associations between the *ADH1B*/*ALDH2* genotypes and cancer risk are shown in Tables 3, 4, 5. We used multivariable regression analysis to assess the risks according to age and genotype in different cancers. The CC genotype of *ADH1B* rs1229984 was significantly associated with cancer of larynx, pharynx, and nasal cavities (OR = 1.56, *p* = 0.001, Table 3), cancer of the pancreas (OR = 1.66, *p* = 0.018, Table 4), and cancer of the esophagus (OR = 4.10, *p* < 0.001, Table 5). In comparison, *ALDH2* rs671 was not associated with cancer of the larynx, pharynx, and nasal cavities, or cancer of the pancreas, but was associated with cancer of the esophagus (OR = 1.52, *p* = 0.003, Table 5). In addition, age is the biggest risk factor for cancer. Hence, we assessed the statistical significance of age by cancer via a multiple logistic regression. In the 41–80 years age group, there was a remarkably higher proportion of participants with cancer of the larynx, pharynx, and nasal cavities in both the age of groups 41–60 and 61–80 than the age group of ≤40 years (Table 3). Regarding cancer of the pancreas, the age group of >60 years had a significantly higher risk than the ≤40 years group (Table 4). In terms of cancer of the esophagus, the age group of 61–80 years showed a significantly greater risk than the other age groups (Table 5).

**TABLE 3:** Multivariable logistic regression analyses of the association between *ADH1B* and *ALDH2* polymorphisms in cancer of larynx, pharynx, and nasal cavities

Variable	Model 1			*p* value	Model 2			*p* value	Model 3			*p* value
	OR	95% CI			OR	95% CI			OR	95% CI		
Age (ref = <40)												
41–60	3.58	2.52	5.09	<0.0001	3.59	2.52	5.10	<0.0001	3.59	2.53	5.10	<0.0001
61–80	3.64	2.57	5.17	<0.0001	3.64	2.56	5.17	<0.0001	3.65	2.57	5.18	<0.0001
>80	2.14	1.30	3.52	0.6313	2.14	1.30	3.52	0.63	2.14	1.30	3.53	0.64
rs1229984 (ref = TT genotype)												
TC genotype	1.04	0.88	1.22	0.04					1.04	0.88	1.22	0.04
CC genotype	1.6	1.20	2.01	0.001					1.56	1.20	2.02	0.001
rs671 (ref = GG genotype)												
GA genotype					1.21	1.03	1.41	0.25	1.21	1.03	1.41	0.24
AA genotype					1.17	0.88	1.56	0.63	1.17	0.88	1.56	0.64

**TABLE 4:** Multivariable logistic regression analyses of the association between *ADH1B* and *ALDH2* polymorphisms in cancer of pancreatic

Variable	Model 1			*p* value	Model 2			*p* value	Model 3			*p* value
	OR	95% CI			OR	95% CI			95% CI	95% CI		
Age (ref = <40)												
41–60	1.87	1.00	3.49	0.33	1.86	0.99	3.48	0.33	1.86	1.00	3.48	0.33
61–80	2.99	1.64	5.46	0.009	2.97	1.63	5.43	0.01	2.98	1.63	5.45	0.009
>80	3.79	1.81	7.94	0.005	3.76	1.79	7.88	0.005	3.78	1.80	7.92	0.005
rs1229984 (ref = TT genotype)												
TC genotype	0.90	0.65	1.24	0.04					0.89	0.65	1.24	0.04
CC genotype	1.7	1.03	2.70	0.02					1.66	1.03	2.70	0.018
rs671 (ref = GG genotype)												
GA genotype					0.86	0.63	1.18	0.97	0.86	0.63	1.17	0.97
AA genotype					0.75	0.40	1.40	0.49	0.75	0.40	1.39	0.49

**TABLE 5:** Multivariable logistic regression analyses of the association between *ADH1B* and *ALDH2* polymorphisms in cancer of esophagus

Variable	Model 1			*p* value	Model 2			*p* value	Model 3			*p* value
	OR	95% CI			OR	95% CI			OR	95% CI		
Age (ref = <60)												
61–80	2.49	1.76	3.53	0.002	2.46	1.74	3.49	0.002	2.48	1.75	3.52	0.002
>80	1.68	0.83	3.43	0.85	1.67	0.82	3.40	0.86	1.69	0.83	3.44	0.84
rs1229984 (ref = TT genotype)												
TC genotype	1.58	1.10	2.27	0.15					1.59	1.11	2.27	0.15
CC genotype	4.1	2.60	6.41	<0.0001					4.10	2.61	6.43	<0.0001
rs671 (ref = GG genotype)												
GA genotype					1.51	1.09	2.10	0.004	1.52	1.09	2.11	0.003
AA genotype					0.51	0.20	1.26	0.05	0.51	0.20	1.26	0.051

Finally, Table 6 presents the associations between the *ADH1B* rs1229984 and *ALDH2* rs671 variants and the risk of cancer. Compared to the wild‐type subjects, participants carrying the rs1229984 TC/CC and rs671 GG genotypes had a higher risk of esophageal cancer (OR = 3.02, 95% CI: 1.54–5.91, *p* < 0.001). In contrast, the risk increased by 100% for cancer of the larynx, pharynx, and nasal cavities (OR = 2.00, 95% CI: 1.41–2.85, *p* < 0.001), 381% for esophageal cancer (OR = 4.81, 95% CI: 2.65–8.75, *p* < 0.001), and 84% for pancreatic cancer (OR = 1.84, 95% CI: 0.99–3.45, *p* = 0.06) in carriers of the rs1229984 TC/CC and rs671 GA/AA genotypes.

**TABLE 6:** Association between *ADH1B* and *ALDH2* polymorphisms and the risk of cancers

Variable	rs1229984_TT & rs671_GA/AA(*n* = 11,244)			*p* value^a^	rs1229984_TC/CC & rs671_GG(*n* = 1585)			*p* value^a^	rs1229984_TC/CC & rs671_GA/AA(*n* = 1415)			*p* value^a^
	OR	95% CI			OR	95% CI			OR	95% CI		
Age (ref = <40)												
41–60	1.00	0.93	1.08	0.81	0.95	0.82	1.11	0.33	1.05	0.89	1.24	0.63
61–80	0.98	0.91	1.06	0.41	0.83	0.71	0.97	0.07	1.05	0.89	1.23	0.67
>80	1.01	0.89	1.14	0.78	0.86	0.66	1.11	0.51	1.00	0.77	1.31	0.81
Gender (ref = Female)												
Male	0.99	0.94	1.04	0.57	0.95	0.86	1.06	0.36	0.93	0.83	1.03	0.17
Cancer of larynx, pharynx, and nasal cavities	1.19	0.96	1.47	0.11	1.41	0.96	2.08	0.08	2.00	1.41	2.85	0.0001
Cancer of esophagus	0.92	0.54	1.55	0.74	3.02	1.54	5.91	0.001	4.81	2.65	8.75	<0.0001
Cancer of pancreatic	0.75	0.50	1.13	0.17	1.09	0.52	2.30	0.82	1.84	0.99	3.45	0.06

*Note*: Control is *ADH1B* rs1229984 and *ALDH2* rs671 wild‐type (*n* = 11,899).

^a^ Using a univariable logistic regression to compare variables between *ADH1B* rs1229984 and *ALDH2* rs671 variants.

## 4 DISCUSSION

In this hospital‐based case–control study, we determined the importance of the *ADH1B* rs1229984 C allele and *ALDH2* rs671 A allele with regard to their association with a high risk of cancer of the larynx, pharynx, hypopharynx and nasal cavities, esophagus, and pancreas. Carriers with the both *ADH1B* rs1229984 TC/CC and *ALDH2* rs671 GA/AA genotypes had a higher risk of cancer than those with the both *ADH1B* rs1229984 TT and *ALDH2* rs671 GA/AA genotypes. The *ADH1B* rs1229984 C allele was found to be significantly associated with alcohol‐related cancers in the Taiwanese population. We also found that genetic factors might contribute to age‐related head and neck cancer. Our results suggested that genetic evaluation of patients with alcohol‐related cancer might provide additional preventive information.

In the present study, the *ADH1B* rs1229984 CC genotype was also found to be significantly associated with alcohol‐induced mental disorders (OR = 2.93, *p* = 0.002) and alcohol dependence syndrome (OR = 3.95, *p* < 0.001). In contrast, the notably decreased risks of alcohol‐induced mental disorders (OR = 0.13), alcohol dependence syndrome (OR = 0.17), and alcohol abuse (OR = 0.34) were statistically significant in the *ALDH2* rs671 carriers (*p* < 0.001). Our finding was consistent with that of a previous study by Yokoyama et al., who reported that individuals with the ADH1B*2 (+) genotype (*1/*2 or *2/*2) and ALDH2*2 (+) genotype (*1/*2 or *2/*2) are likely to suffer from alcohol flushing, preventing alcohol use disorder.^24^ It is worth noting that the protective effect of the *ALDH2* rs671 GA/AA genotypes against alcohol abuse may be a predictive factor of certain psychosocial issues. The Diagnostic and Statistical Manual of Mental Disorders Third Edition (DSM‐III), published in 1980, introduced the nondependence diagnosis of alcohol abuse, recognizing that major problems, for example, alcohol‐related driving felonies and domestic violence, may arise in the absence of alcohol dependence.^30^, ^31^ Additionally, the finding that the risk of cancer of the larynx, pharynx, and nasal cavities is associated with the *ALDH2* rs671 GA/AA genotypes (OR = 1.19, *p* = 0.02) may be explained by the habit of alcohol consumption among these individuals. It is well known that the *ALDH2* rs671 GA/AA genotypes may lead to a higher risk of head and neck cancer with alcohol ingestion.^32^

*ADH1B* rs1229984 and *ALDH2* rs671 are common alcohol metabolism‐related genetic variants in East Asians. By ADH1B and ALDH2, ethanol is metabolized to acetaldehyde and in turn acetaldehyde is converted to acetate. The T allele (major allele among East Asians) of *ADH1B* rs1229984 and A allele (minor allele among East Asians) of *ALDH2* rs671 have been shown to be related to the levels of acetaldehyde accumulated after alcohol ingestion.^33^ *ADH1B* rs1229984 is a missense variant (R48H) located on exon 3 and catalytically oxidizes ethanol into acetaldehyde. Individuals with the rs1229984 His variant (T allele) metabolize ethanol to acetaldehyde 70‐ to 80‐fold faster than individuals with the Arg variant (G allele) due to increased enzymatic function.^20^ Acetaldehyde is subsequently metabolized into harmless acetate, chiefly by *ALDH2*. The *ALDH2* rs671 variant is caused by a missense mutation (E504K). The change in the G allele (Glu504) is changed to the A allele (Lys504) in exon 12 dramatically decreases the enzyme activity of *ALDH2*, leading to a rapid increase in the concentration of acetaldehyde in the blood after alcohol consumption.^34^ This rapid increase in accumulation of acetaldehyde leads to the well‐known Asian alcohol flushing syndrome and has a protective effect against alcohol consumption due to the unpleasant feeling of facial flushing, palpitation, and nausea.^35^, ^36^ The levels of acetaldehyde accumulated were affected by enzyme activities of ADH1B and ALDH2, which are involved in alcohol metabolism pathways, after alcohol consumption. Our finding was consistent with a previous study reporting that the *ADH1B* rs1229984 CC genotype was associated with a 4.02‐fold increased risk of esophagus cancer relative to the heterozygous CT genotype at 1.58‐fold.^37^ Regarding cancers of other origins, a 1.65‐fold increase for pancreatic cancer and a 1.55‐fold increase for head and neck cancer were reported.^38^ We found that the C allele of *ADH1B* rs1229984 is an important risk factor for esophageal cancer, which was significantly promoted by the interaction of the *ADH1B* rs1229984 TC/CC genotype and *ALDH2* rs671 GA/AA genotypes. These results were consistent with the report of Hashibe et al.*, who* reported the *ADH1B* rs1229984 variant as a risk factor for esophageal cancer in the European and Latin American populations.^39^ They are also consistent with the results of another study investigated by Tanaka and his colleagues in a Japanese population.^40^ Two meta‐analyses investigating the effect of genetic variations in *ADH1B* His47Arg (T/C) also showed these to be susceptible loci for esophageal cancer.^37^, ^41^, ^42^ Furthermore, other studies have also provided evidence of a synergistic effect of *ALDH2* rs671 in esophageal cancer.^43^ They reported strong correlations between ALDH2*2/*2 (*ALDH2* rs671 AA genotype) and cancers of the oropharynx and esophagus. Huang et al. also reported that the slow *ADH1B* and slow/nonfunctional *ALDH2* genotypes combination is a risk factor for head and neck cancers.^44^ Nevertheless, in another study, head and neck cancer patients with the fast *ADH1B* and the slow/nonfunctional *ALDH2* genotypes had the poorest overall survival.^45^ *These studies indicated that* fast or slow *ADH1B* have different mechanisms in terms of affecting the patient's cancer risk. Individual with the fast *ADH1B* genotype, the head and neck cancer risk associated with drinking alcohol was raised as compared with those with the slow/nonfunctional *ALDH2* genotypes. For those with the slow *ADH1B* genotypes, oral cleanliness seemed to play a critical role.^46^ The true effects of *ADH1B* and *ALDH2* gene SNPs on alcohol metabolism are debated. When studying alcohol‐related cancers, it might be necessary to consider the synergic effects of alcohol, tobacco and betel‐quid, and condemned mucosa/field cancerization, which complicate the effects of gene polymorphisms.

Our study also demonstrated the effect of *ADH1B* rs1229984 on the risk of pancreatic cancer, especially the CC genotype. However, the association between pancreatic cancer and alcohol consumption remains controversial. Researchers have hypothesized the relationships between pancreatic cancer and alcohol ingestion, and the results are conflicting, especially at low degrees of alcohol consumption.^47^, ^48^, ^49^ Regardless of whether studies report a positive relationship between low/moderate degrees of pancreatic disease and alcohol consumption, individuals who are hereditarily vulnerable to the cancer‐causing impact of alcohol could have a higher risk of pancreatic malignant growth subsequent to drinking alcohol. Similarly, Kanda et al. indicated that the highest pancreatic cancer risk was observed among ever drinkers who carried the *ADH1B* rs1229984 TT genotype in combination with *ALDH2* rs671 GA or *ALDH2* rs671 AA genotype.^50^ In a Taiwanese study, Shan et al.^27^ reported no significant relationship between pancreatic cancer and alcohol consumption, even after considering the level of alcohol consumption and the influences of *ADH1B* and *ALDH2* polymorphisms. However, the relationship between the level of alcohol consumption and the risk of cancer is complex, and the specific nature and impacts of these variants actually need further study. In the present study, the age of the patients was an important factor that reflected the cumulative exposure time to carcinogenic factors in cancers, especially in head and neck cancer. The 41–80 years age group had a significantly higher proportion of participants with cancer of the larynx, pharynx, and nasal cavities than the ≤40 years age group. This finding was consistent with a previous study reporting that the median age of diagnosis for nonviral‐associated HNSCC was 66 years.^51^ Regarding pancreatic cancer, most people who develop this cancer are older than 60. The age group >60 years had a significantly higher risk of pancreatic cancer than the ≤40 years group, and the risk of developing pancreatic cancer increased with age. The odds ratio of age was higher than those for the rs1229984 or rs671 polymorphisms, showing that the causes of cancers are complicated.

In our study, examination of the PheWAS data also implied associations of the*ADH1B* and *ALDH2* variants with alcohol‐related disorders. We found that the *ADH1B* rs1229984 CC genotype increased the risk of alcohol‐induced mental disorders by 193% and of alcohol dependence syndrome by 295%. Specifically, *ADH1B* rs1229984 had a significantly greater impact on problematic drinking among individuals who carried the CC genotype than among those who carried the TC and TT genotypes. On the contrary, the *ALDH2* rs671 GA/AA genotypes significantly reduced the risk of alcohol‐induced mental disorders by 87%, alcohol dependence syndrome by 83%, and alcohol abuse by 66%. These carriers have lower ALDH2 enzymatic activity, and this deficiency is manifested by a greater level of unfavorable symptoms of flushing reactions associated with alcohol consumption; as mentioned above people carrying these alleles will generally stay away from alcohol consumption.^19^, ^36^, ^52^ The combination of *ADH1B* and *ALDH2* therefore has a direct impact on alcohol metabolism and consumption. Since acetaldehyde accumulation induces an unpleasant effect on the body, people carrying the *ALDH2* rs671 A allele are less likely to drink and therefore have a lower risk of alcohol dependence.

There were some limitations of our study. First, it was a case–control study based on hospital population; thus, selection bias might be unavoidable and the subjects may not be delegated to the general population. Second, the genetic polymorphisms examined depended on functional considerations, and they therefore may not give a complete perspective on the hereditary changeability in *ADH1B* and *ALDH2* genes. Third, we lacked data on alcohol intake from each study subject. Some of the risk association may change when alcohol consumption data are taken into consideration. The only information we had was the percentage of those who had ever consumed alcohol (current‐ or ever‐drinker) in our study which was 35.9%. This may have resulted in an underestimation of the magnitude of the genetic susceptibility to carcinogenicity from alcohol use. A previous study showed that alcohol consumption cessation was associated with a 2% yearly reduction in the risk of developing laryngeal and pharyngeal cancers.^53^ The report shows that the epigenetic effects promoted by alcohol seem to retroact with abstinence. Therefore, larger, well‐designed studies with alcohol consumption data are required to confirm our findings. Finally, the clinical diagnoses in our study were based on ICD‐9‐CM not its current version. We could not obtain itemized data on cancer metastasis, which limited further investigations of the roles of *ADH1B* and *ALDH2* polymorphisms in the cancer progression and prognosis.

## 5 CONCLUSION

This hospital‐based study demonstrated that *ADH1B* rs1229984 appears to have a stronger effect on alcohol‐related disorders and simultaneously is associated with a higher risk of alcohol‐related cancers. Our data showed that participants carrying *ADH1B* rs1229984 TC/CC and *ALDH2* rs671 GA/GG were at higher risk of esophagus cancer than noncarriers. This result suggests that a decrease in alcohol ingestion is important for high‐risk patients with the *ADH1B* rs1229984 C allele.

## AUTHOR CONTRIBUTIONS

TGC, TTY, CYW, THH, and ICC wrote the manuscript. TTY, CYW, and ICC analyzed the data. TGC, TTY, and THH performed clinical analysis. TGC, THH, and ICC designed the study. All the authors read and approved the final version of the manuscript.

## CONFLICT OF INTEREST

The authors declare no conflict of interest.

## ETHICS STATEMENT

This study involving human participants were approved by the ethics committee of Taichung Veterans General Hospital Institutional Review Board (IRB no. SF19153A).

## Data Availability

The datasets analyzed during this study are included in this publication.
